# Relationship between clinical nurses’ job satisfaction and emotional regulation: the mediating role of positive affect

**DOI:** 10.3389/fpsyg.2026.1922739

**Published:** 2026-10-07

**Authors:** Wen Liu, Yalan Li, Jili Zeng, Ying Deng

**Affiliations:** Department of Nursing, General Hospital of Central Theater Command of the Chinese People's Liberation Army, Wuhan, Hubei, China

**Keywords:** emotional regulation, job satisfaction, nurses, positive affect, professional benefit perception

## Abstract

**Objective:**

To investigate the relationships among nurses’ emotional regulation, positive affect, professional benefit perception, and job satisfaction, with a particular focus on the mediating role of positive affect.

**Methods:**

A cross-sectional descriptive study was conducted among nurses from three mainland Chinese hospitals. Data from 1,226 nurses were collected using validated scales: the Nurse Job Satisfaction Scale, Nurse Emotional Regulation Scale, Professional Benefit Perception Scale, and Positive and Negative Affect Schedule (PANAS). Statistical analyses were performed using SPSS 27.0 and the PROCESS macro to test the hypothesized model.

**Results:**

Nurses’ emotional regulation and positive affect were significantly and positively associated with job satisfaction. Professional benefit perception was also independently and positively associated with job satisfaction alongside emotional regulation. However, no significant moderating effect of professional benefit perception was observed on the relationship between emotional regulation and job satisfaction.

**Conclusion:**

Emotional regulation was positively associated with job satisfaction, and positive affect partially mediated this association in the tested statistical model. Professional benefit perception showed an independent positive association with job satisfaction but did not significantly moderate the association between emotional regulation and job satisfaction. Longitudinal research is needed to clarify temporal and causal relationships.

**Implications for nursing management:**

This study provides empirical evidence from China’s healthcare system, clarifying the interplay between emotional regulation and job satisfaction. The findings may inform the development and future evaluation of interventions targeting nurses’ psychological resources and workplace experiences.

## Introduction

Among healthcare professionals globally, nurses are recognized to experience elevated levels of work-related stress and mental health risks due to their multifaceted roles and exposure to occupational pressures including frequent assessments and public health emergencies (McCloughen et al., 2020; Quan et al., 2023). Nurses working in high-stress environments not only demonstrate increased susceptibility to fatigue and negative emotional states, but also exhibit significantly higher prevalence of anxiety, stress, depression, sleep disturbances, and emotional exhaustion. A multinational study across 13 regions revealed that nurses were three times more likely than the general population to screen positive for depression (OR = 3.02) and anxiety (OR = 2.92), with those experiencing sleep difficulties showing 15-fold higher odds of positive depression screening (Norful et al., 2024). Such negative emotional states not only impair nurses’ physical and psychological well-being but also undermine professional identity, obscure occupational fulfillment, and precipitate emotional exhaustion, ultimately leading to diminished job satisfaction.

Existing research demonstrates strong correlations between nurses’ emotional regulation capacity and critical outcomes including job satisfaction, occupational well-being, and mental health (Zhou et al., 2024; Yu et al., 2023). Poor emotional control frequently contributes to communication breakdowns in nurse–patient and interprofessional relationships, thereby negatively impacting both job satisfaction and care quality (Cao et al., 2024).

Emotional regulation refers to the psychological process of modulating individual or collective emotional states through systematic strategies, aimed at mitigating negative affect while maintaining appropriate emotional and behavioral responses (Zhu et al., 2022). Nurses’ emotional regulation capacity demonstrates significant associations with mental health status, job satisfaction, and professional fulfillment (Chen et al., 2022). The theoretical framework of this study is grounded in the Job Demands-Resources (JD-R) model, which posits that job demands (e.g., emotional labor, workload) consume energy and lead to strain, while job resources (e.g., emotional regulation skills, social support) foster motivation and engagement (Bakker and Demerouti, 2024). Within this framework, emotional regulation can be conceptualized as a personal resource that buffers the negative effects of job demands and enhances job satisfaction. Furthermore, Fredrickson’s (2001) Broaden-and-Build Theory provides a complementary lens: positive emotions broaden individuals’ cognitive and behavioral repertoires and build enduring psychological resources, which may explain why emotional regulation indirectly enhances job satisfaction through the cultivation of positive affective states. The ABC theory of emotions posits that negative emotional responses (Consequence) arise not directly from activating events (Activating event), but rather from individuals’ irrational cognitions and beliefs (Beliefs) regarding such events, with positive cognitive appraisals generating constructive emotional outcomes (Liu et al., 2025). Professional benefit perception, an emerging concept in positive psychology, denotes nurses’ recognition and internalization of career-derived personal gains and advantages during professional practice (Hu et al., 2020), representing a positive cognitive evaluation and affective experience toward their vocation. While reducing negative emotions remains a common strategy for addressing health and behavioral issues, enhancing positive emotions has emerged as an equally vital approach for health promotion.

International evidence has further elucidated the mechanisms linking emotion regulation to occupational well-being in healthcare workers. Di Gesto et al. (2025) demonstrated that psychological inflexibility fully mediated the relationship between emotion regulation difficulties and perceived stress among ICU nurses, highlighting the importance of psychological flexibility as a novel construct in understanding how emotion regulation influences stress outcomes in healthcare professionals. Similarly, Hemade et al. found that work-life balance mediated the relationship between emotional intelligence and job satisfaction among critical care nurses, suggesting that emotional competencies operate through multiple pathways to influence occupational satisfaction (Hemade et al., 2025). These studies underscore the need for a more nuanced theoretical account of how emotional regulation—directly and indirectly—shapes nurses’ job satisfaction.

In the present study, we propose the following explicit hypotheses based on the integrated theoretical framework (JD-R model and Broaden-and-Build Theory):

*H1*: Emotional regulation is positively associated with job satisfaction.

*H2*: Positive affect mediates the association between emotional regulation and job satisfaction.

*H3*: Professional benefit perception moderates the association between emotional regulation and job satisfaction, such that the association is stronger when professional benefit perception is higher.

We aim to investigate the mediating role of positive emotions in the relationship between nurses’ emotional regulation and job satisfaction, along with the moderating effect of professional benefit perception on job satisfaction. The findings may inform the development and evaluation of stress-reduction and psychological-support strategies that foster adaptive emotional regulation and positive affect. Ultimately, this research seeks to promote mental health preservation among nursing professionals across diverse healthcare settings.

## Materials and methods

### Participants

From January to March 2025, a convenience sample of clinical nurses was recruited from three general hospitals across mainland China. Eligible participants were registered nurses with at least 1 year of clinical experience who were working in frontline roles; those in administrative or logistical positions were not included. Nurses were excluded if they were on leave or engaged in external training programs during the study period.

This study received ethical approval from the Institutional Review Board of the researchers’ hospital ([2025]075–01). All procedures performed were in accordance with the ethical standards of the institutional research committee. Informed consent was obtained from all individual participants included in the study.

### Questionnaire introduction

The following instruments were used in this study:

(1) Demographic Questionnaire

A self-developed questionnaire was designed by the research team based on a literature review and the study objectives. It comprised 18 items covering workplace, department, gender, age, education level, professional title, years of work experience, personality traits, night-shift frequency, knowledge of emotional regulation, prior training in emotional regulation, stress-coping strategies, and overall emotional state during work.

(2) Nurse Emotional Regulation Scale (NERS) (Fu et al., 2014)

The NERS is an 18-item instrument assessing emotional regulation across four dimensions: emotional awareness, emotional expression, emotional adjustment, and emotional utilization. Items were rated on a 5-point Likert scale ranging from 1 (“strongly disagree”) to 5 (“strongly agree”), with higher scores indicating greater emotional regulation ability. The original scale demonstrated a Cronbach’s α of 0.835 and a test–retest reliability of 0.743. In the present study, Cronbach’s α was 0.792.

(3) Nurse Job Satisfaction Scale (NJSS) (Tao et al., 2009)

The NJSS is a 38-item scale adapted from the Mueller/McCloskey Satisfaction Scale (MMSS). It assesses job satisfaction across eight domains: management, workload, relationships with colleagues, job content, compensation, professional growth, recognition, and work-life balance. Each item was scored on a 5-point Likert scale ranging from 1 (“fully disagree”) to 5 (“fully agree”), with higher total scores reflecting greater job satisfaction. The original scale reported a Cronbach’s α of 0.815 and a test–retest reliability of 0.852. In the current study, Cronbach’s α was 0.880.

(4) Positive and Negative Affect Schedule (PANAS) (Huang et al., 2003)

The PANAS is a 20-item Chinese adaptation that measures positive affect (10 items) and negative affect (10 items). The original validation study reported a Cronbach’s α of 0.82 for the total scale. In this study, Cronbach’s α was 0.936.

(5) Professional Benefit Perception Questionnaire (Hu et al., 2020)

A simplified 17-item version of this questionnaire was used to assess nurses’ perceptions of professional benefits across five dimensions: positive career perception (3 items), nurse–patient relationships (4 items), social recognition (3 items), team belonging (3 items), and personal growth (4 items). Items were rated on a 5-point Likert scale ranging from 1 (“strongly disagree”) to 5 (“strongly agree”), with total scores ranging from 17 to 85. The original subscales demonstrated Cronbach’s α coefficients ranging from 0.74 to 0.85, and split-half reliability coefficients ranging from 0.674 to 0.877. In the present study, Cronbach’s α for the overall scale was 0.963.

### Data collection

Approval was obtained from nursing department leaderships. Online surveys included informed consent documentation and restricted single IP submissions. Data from Wenjuanxing platform were exported to SPSS. Invalid responses (completion time<3 or>30 min, patterned/logically inconsistent answers) were excluded. Of 1,380 collected questionnaires, 1,226 were valid (valid response rate: 88.84%).

### Statistical analysis

Data were analyzed using Excel 2019 and SPSS 27.0. Continuous variables were presented as mean ± standard deviation (SD), and categorical variables as frequencies and percentages. Prior to regression analyses, we verified the assumptions of linear regression: normality via Q-Q plots and Shapiro–Wilk tests, homoscedasticity via scatterplots of standardized residuals against standardized predicted values, multicollinearity (variance inflation factor < 10 for all predictors), and linearity via partial regression plots; all assumptions were satisfied.

Pearson correlation analysis was used to examine bivariate relationships, and hierarchical regression analysis was employed to test the moderating effect of professional benefit perception. For mediation analysis, we used the PROCESS macro (Model 4) with 5,000 bootstrap resamples to test the mediating role of positive affect in the relationship between emotional regulation and job satisfaction. Because the study uses cross-sectional data, the mediation model should be interpreted as testing statistically consistent associations rather than proving causal pathways; causal inferences cannot be drawn from these data. In both the mediation and moderation analyses, we controlled for demographic variables that showed significant associations with job satisfaction in preliminary analyses (age, professional title, marital status, and having children) to provide a more rigorous evaluation of the proposed model.

The significance level was set at *α* = 0.05. Bias-corrected 95% confidence intervals (CIs) for indirect effects were obtained from the bootstrap procedure, whereas CIs for direct effects and moderation coefficients were derived from the t-distribution, which yields virtually identical estimates to bootstrap CIs given the large sample size (*N* = 1,226).

## Results

### Status of clinical nurses’ job satisfaction

This study included 1,226 clinical nurses. Their job satisfaction score was (134.47 ± 14.49) points, indicating a moderate level. Scores for specific dimensions are detailed in Table 1.

**Table 1:** Variables and scores (*N* = 1,226).

Variables	Average value	Standard deviation
Job satisfaction	134.47	14.49
Management	25.43	4.30
Workload	25.90	2.94
Colleague relationship	17.95	2.07
Work itself	16.92	2.64
Personal growth and development	13.24	2.67
Recognition	13.60	2.07
Work-family balance	14.29	1.91

The analysis of differences in job satisfaction revealed significant differences based on whether nurses had children, age, marital status, professional title, and family relationships, as shown in Table 2, which also clearly provides the statistical test results (t/F and *p*-values) for each category.

**Table 2:** Differences in job satisfaction across demographic characteristics (*N* = 1,226).

Variable	Sort	M ± SD	t/F	*p*
Sex	Male	3.64 ± 0.46	1.69 (t)	0.09
	Female	3.52 ± 0.37		
Have children	Yes	3.56 ± 0.38	3.58 (t)	<0.001
	No	3.47 ± 0.36		
Age	<25	3.50 ± 0.32	6.02 (F)	<0.001
	25–29	3.48 ± 0.43		
	30–34	3.48 ± 0.35		
	35–39	3.54 ± 0.37		
	40–45	3.62 ± 0.37		
	>45	3.75 ± 0.36		
Marital status	Married	3.55 ± 0.38	4.00 (F)	<0.001
	Unmarried	3.48 ± 0.36		
	Other	3.48 ± 0.39		
Educational background	Technical secondary school	3.49 ± 1.08	0.18 (F)	0.91
	Junior college	3.52 ± 0.35		
	Bachelor’s degree	3.52 ± 0.37		
	Master’s degree or above	3.61 ± 0.29		
Professional title	Nurse	3.52 ± 0.41	3.56 (F)	0.01
	Nurse practitioner	3.48 ± 0.35		
	Supervisor nurse	3.55 ± 0.38		
	Associate chief nurse and above	3.69 ± 0.33		
Family relationship	Good	3.54 ± 0.36	11.04 (F)	<0.001
	Average	3.40 ± 0.40		
	Poor	4.06 ± 1.32		

### Correlations among job satisfaction, emotional regulation, positive affect, and professional benefit perception

Pearson correlation analysis revealed that job satisfaction and its dimensions were significantly positively correlated with other key variables, as detailed in Table 3. For instance, job satisfaction showed significant positive correlations with emotional regulation (*r* = 0.53, *p* < 0.01), positive affect (*r* = 0.44, *p* < 0.01), and professional benefit perception (*r* = 0.69, *p* < 0.01).

**Table 3:** Correlation matrix of study variables (*N* = 1,226).

Variables	1	2	3	4	5	6	7	8	9	10	11	12
1 Management	1.00											
2 Workload	0.42**	1.00										
3 Colleague relationship	0.61**	0.53**	1.00									
4 Work itself	0.18**	0.39**	0.30**	1.00								
5 Wages and benefits	0.60**	0.32**	0.49**	0.17**	1.00							
6 Personal growth and development	0.61**	0.47**	0.53**	0.33**	0.48**	1.00						
7 Recognition	0.54**	0.53**	0.57**	0.37**	0.44**	0.56**	1.00					
8 Work-family balance	0.58**	0.28**	0.55**	0.16**	0.46**	0.43**	0.42**	1.00				
9 Emotional regulation	0.39**	0.45**	0.47**	0.29**	0.30**	0.41**	0.41**	0.36**	1.00			
10 Job satisfaction	0.83**	0.70**	0.79**	0.50**	0.70**	0.77**	0.75**	0.65**	0.53**	1.00		
11 Positive affect	0.42**	0.23**	0.38**	0.10**	0.35**	0.35**	0.34**	0.33**	0.29**	0.44**	1.00	
12 Professional benefit perception	0.64**	0.48**	0.63**	0.19**	0.46**	0.49**	0.49**	0.50**	0.43**	0.69**	0.41**	1.00

*p < 0.05, **p < 0.01.

### The mediating role of positive affect in the association between emotional regulation and job satisfaction

Regression analysis demonstrated that nurses’ emotional regulation was significantly and positively associated with job satisfaction (Table 4). Bootstrap analysis was used to estimate the indirect statistical association. The total effect of emotional regulation on job satisfaction was 0.9808 (SE = 0.0445, *p* < 0.001, 95% CI [0.8935, 1.0682]). After controlling for the mediating variable (positive affect), the direct effect remained significant (B = 0.8173, SE = 0.0435, *p* < 0.001, 95% CI [0.7319, 0.9027]), while the indirect effect of emotional regulation through positive affect was 0.1636 (SE = 0.0237, 95% CI [0.1175, 0.2166]), accounting for 16.68% of the total effect. These results are consistent with partial mediation in the tested statistical model; however, as the data are cross-sectional, this mediation should be interpreted as a statistical association consistent with the hypothesized pathway, not as evidence of causation. A visual depiction of the tested mediation model with standardized coefficients is provided in Figure 1.

**Table 4:** Mediation analysis (*N* = 1,226).

Outcome	Predictor/statistic	B	SE	*t*	*p*	*β*	95% CI
Job satisfaction (total effect)	Constant	69.766	2.957	23.594	<0.001***	-	[64.01, 75.52]
	Emotional regulation	0.981	0.045	22.038	<0.001***	0.533	[0.8935, 1.0682]
	R^2^ = 0.284; adjusted R^2^ = 0.283; *F*(1, 1,224) = 485.686; *p* < 0.001						
Positive affect (path a)	Constant	9.852	1.891	5.209	<0.001***	-	[6.14, 13.56]
	Emotional regulation	0.299	0.028	10.501	<0.001***	0.287	[0.244, 0.354]
	R^2^ = 0.083; adjusted R^2^ = 0.081; *F*(1, 1,224) = 110.262; *p* < 0.001						
Job satisfaction (direct effect)	Constant	64.375	2.802	22.978	<0.001***	-	[58.88, 69.87]
	Emotional regulation	0.817	0.044	18.768	<0.001***	0.444	[0.7319, 0.9027]
	Positive affect	0.547	0.042	13.066	<0.001***	0.309	[0.465, 0.629]
	R^2^ = 0.372; adjusted R^2^ = 0.370; *F*(2, 1,223) = 361.867; *p* < 0.001						
Indirect effect	Emotional regulation → Positive affect → Job satisfaction	0.1636	0.0237	-	-	-	[0.1175, 0.2166]

*p < 0.05, **p < 0.01, ***p < 0.001.

**Figure 1:**
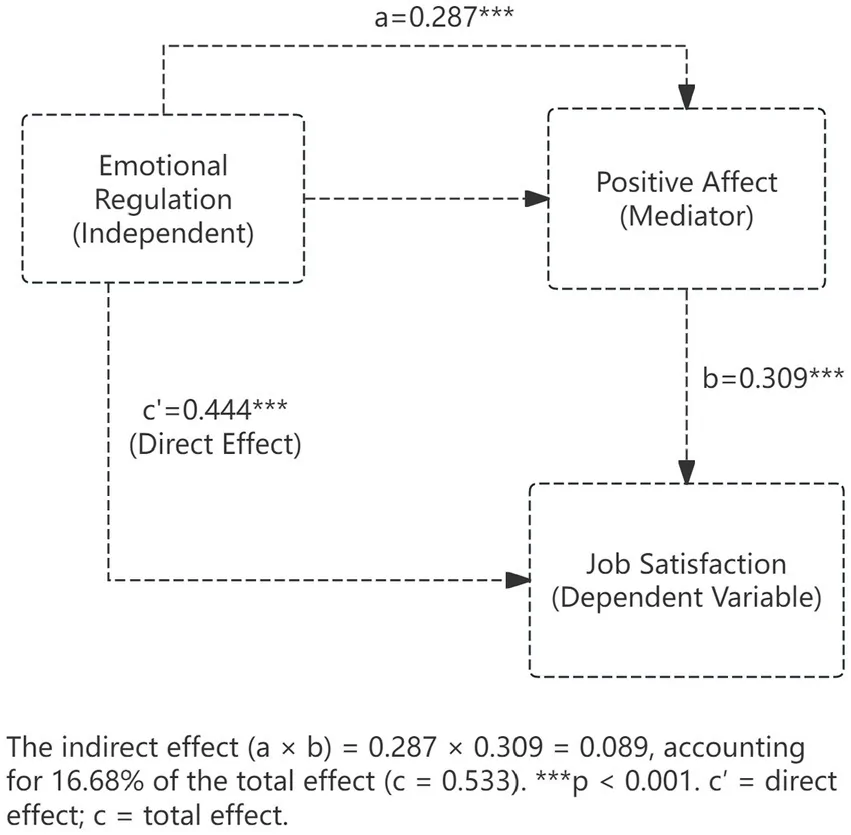
Mediation model of the association between emotional regulation and job satisfaction through positive affect. Values shown are standardized regression coefficients (β).

### The moderating effect of professional benefit perception on the association between emotional regulation and job satisfaction

The results showed that both nurses’ emotional regulation and professional benefit perception independently exerted significant positive associations with job satisfaction. However, professional benefit did not demonstrate a significant moderating effect on the relationship between emotional regulation and job satisfaction, moderation effect: B = −0.002, SE = 0.002, *t* = −1.068, *p* = 0.286, *β* = −0.171, as shown in Table 5.

**Table 5:** Hierarchical regression analysis of the moderating effect (*N* = 1,226).

Model	Predictor/statistic	B	SE	*t*	*p*	*β*	95% CI
Step 1	Constant	69.766	2.957	23.594	<0.001***	-	[64.01, 75.52]
	Emotional regulation	0.981	0.045	22.038	<0.001***	0.533	[0.8935, 1.0682]
	R^2^ = 0.284; adjusted R^2^ = 0.283; *F*(1, 1,224) = 485.686; *p* < 0.001						
Step 2	Constant	41.475	2.604	15.926	<0.001***	-	[36.37, 46.58]
	Emotional regulation	0.538	0.039	13.618	<0.001***	0.292	[0.462, 0.614]
	Professional benefit perception	0.841	0.032	26.151	<0.001***	0.561	[0.778, 0.904]
	R^2^ = 0.541; adjusted R^2^ = 0.540; *F*(2, 1,223) = 720.278; *p* < 0.001						
Step 3	Constant	30.609	10.506	2.914	0.004***	-	[10.02, 51.20]
	Emotional regulation	0.705	0.162	4.363	<0.001***	0.383	[0.3875, 1.0225]
	Professional benefit perception	1.005	0.156	6.422	<0.001***	0.670	[0.6992, 1.3108]
	Emotional regulation × Professional benefit perception	-0.002	0.002	−1.068	0.286	−0.171	[−0.0059, 0.0019]
	R^2^ = 0.541; adjusted R^2^ = 0.540; *F*(3, 1,222) = 480.620; *p* < 0.001						

*p < 0.05, **p < 0.01, ***p < 0.001.

## Discussion

### Clinical nurses’ job satisfaction at a moderate level

The findings of this study indicate that clinical nurses’ job satisfaction is at a moderate level. Among the dimensions evaluated, the lowest score was observed in “personal growth and development” (13.24 ± 2.67).

This aligns with prior research by Zhao et al. (2021) and Zhang et al. (2025), which reported a generally low-to-moderate job satisfaction level among clinical nurses in China.

A meta-analysis further highlighted regional and sampling-based disparities in nurses’ job satisfaction (Liu et al., 2024). For instance, specialty nurses exhibit higher teamwork satisfaction compared to general nurses (Zhao et al., 2025). This phenomenon may stem from chronic exposure to high-intensity workloads, complex patient-provider relationships, and shift work—stressors that contribute to elevated emotional exhaustion and compromised nursing quality and safety.

These findings resonate with Aiken’s et al. (2014) “Triple Threat in Nursing” theory, which underscores the interplay of workforce shortages, unsafe working conditions, and burnout within the broader context of the Job Demands-Resources model, wherein high job demands coupled with insufficient resources deplete nurses’ energy and undermine satisfaction (Bakker and Demerouti, 2024).

Additionally, this study identified significant correlations between nurses’ job satisfaction and individualized factors such as parental status (e.g., having children), professional rank, and family relationship quality. Nurses aged 45 or older reported higher satisfaction than those under 39. Satisfaction levels also varied by professional rank: staff nurses exhibited significantly lower satisfaction compared to nurse-in-charge and associate chief nurses. Intriguingly, nurses with poor family relationships reported higher job satisfaction than those with good or average family relationships—a counter-intuitive finding that warrants deeper psychological interpretation. We note that the following explanations for this pattern are tentative and speculative, requiring confirmation in future longitudinal research.

First, nurses raising children may experience heightened work–family conflict, particularly under shift schedules and irregular working hours, where parenting demands exacerbate physical and psychological strain, thereby diminishing job satisfaction (Tang et al., 2025).

Second, disparities in professional rank likely influence satisfaction through career advancement opportunities and job autonomy. Higher-ranking nurses often engage in managerial roles and decision-making, fostering a stronger sense of professional fulfillment, whereas lower-ranking nurses may face burnout due to repetitive tasks and limited promotion pathways (Aiken et al., 2013). Third, family relationships, as a critical source of social support, may buffer the adverse psychological effects of workplace stress. Harmonious family dynamics can provide emotional resources to help nurses navigate professional challenges (Zhao et al., 2023). The counter-intuitive finding that nurses with poor family relationships reported higher job satisfaction than those with good or average relationships may reflect a compensatory mechanism, wherein work serves as a psychological buffer or escape from personal challenges. Alternatively, nurses with strained family relationships might invest more emotional energy and time in their professional roles, deriving fulfillment and positive identity from work that compensates for deficits in the family domain. However, given the cross-sectional nature of our data, this interpretation remains speculative and requires longitudinal investigation. These results underscore the need for tailored strategies to enhance nurses’ job satisfaction. Interventions could include implementing flexible scheduling for nurses with childcare responsibilities, optimizing career advancement frameworks, and strengthening social support networks through family-friendly policies. Future research should explore the interactions among these factors and design targeted interventions to address the multifaceted determinants of nurses’ job satisfaction.

### Emotional regulation and perceived professional benefits are positively associated with nurses’ job satisfaction

This study found that emotional regulation and perceived professional benefits were independently associated with nurses’ job satisfaction. First, nurses’ capacity for emotional regulation may support their ability to cope with occupational stressors. Nurses with strong emotional regulation skills exhibit higher emotional intelligence, enabling them to mitigate negative emotions (e.g., anxiety from patient conflicts or high-intensity workloads) through adaptive strategies such as cognitive reappraisal. These processes may be associated with lower burnout and greater psychological resilience and job satisfaction (Hemade et al., 2025). This finding is consistent with Di Gesto et al. (2025) demonstration that emotion regulation difficulties predict perceived stress through psychological inflexibility, underscoring the importance of adaptive emotion regulation processes in healthcare settings.

Second, perceived professional benefits reflect nurses’ internalized recognition of their work’s value, including professional accomplishment, perceived societal contribution, and satisfaction from skill advancement. Studies indicate (Zhan et al., 2020) that nurses with stronger perceived benefits are more likely to reframe challenges as growth opportunities and bolster job engagement through positive self-appraisal.

Emotional regulation may assist nurses in navigating short-term stressors, whereas perceived professional benefits may reinforce longer-term occupational commitment; both were independently associated with job satisfaction (Othman et al., 2024) within the JD-R framework, where personal resources (emotional regulation) and contextual resources (professional benefit perception) may jointly support work engagement and satisfaction (Bakker and Demerouti, 2024). This interpretation is robust as it directly follows from the theoretical framework and is supported by the observed independent main effects.

Healthcare institutions should prioritize targeted emotional regulation training (e.g., mindfulness-based interventions, and stress-management workshops) and strengthen professional development support (e.g., continuing education, and recognition programs) to enhance nurses’ occupational identity. Such systemic interventions can improve workforce stability and care quality by addressing both psychological and professional dimensions of nursing practice.

### Positive emotions mediate the relationship between emotional regulation and job satisfaction in clinical nurses

Regression analysis revealed that emotional regulation was significantly associated with nurses’ job satisfaction when the mediator (positive affect) was excluded (unstandardized coefficient B = 0.981, *t* = 22.038, *p* < 0.001; standardized coefficient = 0.533). This indicates a substantial positive association between emotional regulation and job satisfaction, where stronger emotional regulation skills correspond to higher satisfaction. When positive affect was introduced as a mediator, the association between emotional regulation and job satisfaction remained significant but attenuated (B = 0.817, *β* = 0.444, *t* = 18.768, *p* < 0.001). The significant indirect effect was consistent with partial mediation in the tested statistical model, suggesting that positive affect accounted for part of the association between emotional regulation and job satisfaction. Consistent with our pre-registered caution, this mediation result is statistically consistent with the hypothesized pathway but should not be interpreted as causal evidence given the cross-sectional design.

Aligning with Fredrickson’s (2001) Broaden-and-Build Theory, positive affect expands nurses’ cognitive and behavioral resources (e.g., creativity, problem-solving) and build long-term psychological resilience, enabling them to maintain effectiveness in high-stress environments.

Specifically, nurses with advanced emotional regulation skills strategically mitigate negative emotions (e.g., patient conflict or burnout) and actively cultivate positive affect (e.g., accomplishment, hope), thereby enhancing their sense of control and meaningfulness in work (Bakker and Demerouti, 2024; Yikilmaz et al., 2024).

This accumulation of positive affect not only buffers the adverse psychological impacts of job stress but also strengthens professional identity, further elevating satisfaction (Sun et al., 2022).

For instance, a study on emergency department nurses demonstrated significant mediating effects of positive affect between emotional labor and job satisfaction, with emotion-focused interventions effectively enhancing positive affectivity (Xu et al., 2020).

Healthcare institutions should prioritize emotion-focused training programs (e.g., mindfulness-based stress reduction, peer support groups) to systematically nurture nurses’ emotional regulation skills and amplify positive affect. Additionally, clinical supervision structured to improve clinical skills, knowledge, and professional competence has been shown to enhance job satisfaction, reduce burnout and stress, and improve care quality. Notably, nurses with structural empowerment (e.g., autonomy, access to resources, and decision-making influence) are more likely to hold positive beliefs about their ability to contribute meaningfully in the workplace, correlating with higher job satisfaction compared to those lacking such empowerment (Yesilbas and Kantek, 2024).

### The non-significant moderating role of professional benefit perception

Contrary to our hypothesis (H3), professional benefit perception did not significantly moderate the relationship between emotional regulation and job satisfaction (B = −0.002, SE = 0.002, *t* = −1.068, *p* = 0.286). This null finding warrants careful interpretation. One possible explanation is that emotional regulation and professional benefit perception operate through largely independent pathways to influence job satisfaction. Emotional regulation—as a personal resource—may primarily affect satisfaction through intrapsychic processes (e.g., stress reduction, positive affect enhancement), whereas professional benefit perception—as a contextual evaluation—may influence satisfaction through cognitive appraisal of one’s work environment and professional identity. When both variables are entered simultaneously, their main effects account for a substantial portion of variance in job satisfaction (R^2^ = 0.541), leaving little unique variance for their interaction to explain. This interpretation is consistent with the JD-R model’s distinction between personal resources and contextual resources, which may exert additive rather than multiplicative effects on occupational well-being (Bakker and Demerouti, 2024). Alternatively, the non-significant moderation may reflect measurement issues, such as range restriction or the specific operationalization of professional benefit perception in this study. Future research should explore whether professional benefit perception might be better conceptualized as an independent predictor or mediator rather than a moderator, particularly given its strong direct association with job satisfaction (*β* = 0.561, *p* < 0.001) observed in this study. Given the null result, we emphasize that the absence of moderation is a robust finding in this sample, but the explanations remain tentative and require replication.

## Conclusion

This study demonstrates that emotional regulation among nurses is positively associated with job satisfaction, with this relationship partially mediated by positive affect. However, given the cross-sectional design, these findings reflect correlation rather than causal relationships, and causal inferences should not be drawn. To optimize nursing outcomes, hospital administrators may consider developing and evaluating emotion-regulation and psychological-support strategies for clinical nurses. Longitudinal and intervention studies are needed to determine whether such approaches improve job satisfaction and related workforce outcomes.

### Strengths and limitations

This study has several strengths, including a large sample size (*N* = 1,226) and the use of validated instruments with good reliability in the current sample. The application of PROCESS macro with bootstrap resampling provided robust estimates of indirect effects.

Several limitations should be acknowledged. First, the cross-sectional design precludes causal inferences regarding the relationships among emotional regulation, positive affect, and job satisfaction. The mediation model, while theoretically grounded, is statistically equivalent to alternative models that cannot be distinguished with cross-sectional data. We explicitly remind readers that the mediation results reflect statistical associations consistent with the hypothesized model, not causal pathways. Second, the convenience sampling method may limit the generalizability of findings beyond the three participating hospitals in mainland China. Third, the exclusive reliance on self-report questionnaires introduces the potential for common method variance and social desirability bias, which may have inflated the observed associations. Fourth, the non-significant moderation findings should be interpreted with caution, as they may reflect statistical power limitations or the specific operationalization of constructs. Future longitudinal and experimental studies are needed to establish causal pathways and to examine whether interventions targeting emotional regulation and positive affect can effectively enhance nurses’ job satisfaction across diverse healthcare settings.

## Data Availability

The data supporting the findings of this study are available from the corresponding author upon reasonable request, subject to applicable ethical and institutional requirements.
